# Fishing capacity evaluation of fishing vessel based on cloud model

**DOI:** 10.1038/s41598-022-12852-8

**Published:** 2022-05-28

**Authors:** Chao Lyu, He-xu Zhang, Shuang Liu, Yi Guo

**Affiliations:** 1 College of Engineering Science and Technology, Shanghai Ocean University, Shanghai, 201306 China; 2 Fisheries Bureau, Ministry of Agriculture and Rural Affairs, Beijing, 100125 China

**Keywords:** Mechanical engineering, Marine biology

## Abstract

In the evaluation problem of fishing vessel fishing capacity, the imperfect evaluation index system and the methods of evaluation indexes are mostly artificial qualitative evaluation methods, which lead to strong subjectivity and fuzziness as well as low accuracy of evaluation results. Therefore, this study introduces cloud model theory on the basis of improving the evaluation index system, converts the artificial qualitative evaluation results into the digital characteristics of clouds, realizes the mutual transformation of qualitative evaluation and quantitative evaluation, and improves the accuracy of evaluation results. Taking the trawler as an example, the cloud model method is used to evaluate the fishing capacity, and the result obtained is (77.1408, 1.6897, 0.0), the result obtained by the fuzzy comprehensive evaluation method is 76.664785, and the result obtained by the cloud center of gravity evaluation method is 0.7919. Compared with the other two methods, the cloud model method uses three numerical characteristics to describe the results, and combining the different numerical characteristics meanings, the evaluation results can be judged to be accurate, and the influence of ambiguity on the results is greatly reduced. Meanwhile, the evaluation results can be presented in the form of pictures, and the results are more intuitive; in addition, the cloud model of the evaluation results is compared with the standard cloud model for similarity, which improves the credibility and authenticity of the results.

## Introduction

In 2018, the total output of global fishing industry reached an all-time high of 96.4 million tons, an increase of 5.4% over the average of the previous three years^1^. Fishing capacity refers to the fishing capacity of a ship or a fleet, which is the maximum catch that a fishing vessel (or a fleet) can produce in a certain period (year or season) under the condition of given fishery resources or biomass and under the existing technology, when the ship (or fleet) is fully utilized (that is, the capacity utilization rate reaches 100%). At present, most countries are faced with excess fishing capacity. For example, about 57% of China's marine fish resources were over-exploited or collapsed, and the marine ecosystem was under tremendous pressure due to the development of coastal cities^2^. At the same time, the fishing capacity of octopus in South Korea's coastal waters obtained by data envelopment analysis (DEA) showed that it was necessary to reduce the excess fishing capacity as soon as possible^3^. To deal with the above situation, it is particularly important to study the fishing capacity. At present, the commonly used methods to analyze and measure marine fishing capacity include peak value method (PTP), data envelopment analysis (DEA) and stochastic production boundary method (SPF)^4^. The research on fishing capacity is mainly reflected in the following aspects.

In the aspect of fishery management policy, DEA was used to evaluate the productivity of fishery, which provided help for the follow-up fishery management^5^. But DEA may produce randomness, so a two-stage model could be used to estimate the returns of different inputs to fishery production, the obtained results also provided help for fishery managers^6^. By using DEA to analyze the economic performance and capacity efficiency, it showed that overcapacity was mainly caused by the use of high-tech technology and current policies^7^. To solve the problem of overcapacity, international cooperation in fisheries is very important, and fishers should also participate in supervision^8,9^. In addition, through the analysis of fishers and fishing companies using DEA and endogenous transformation regression model, the degree of overcapacity was obtained, the performance of fishing companies was analyzed to investigate the influence of operators' participation in monitoring on the level of overcapacity^9,10^.

In the aspect of fishing efficiency, DEA was used to analyze the marine fisheries in northeast Spain, and it showed that the unpredictability of resources in fishing operations had the most significant impact on small-scale fishing, which was the main reason for the low efficiency of small-scale fishing^11^. The coastal fisheries in 11 provinces of China were analyzed by stochastic frontier analysis method, and it showed that the main factors affecting technical efficiency were economy and natural environment, industry development and relevant government policies^12^. Measuring the fishing efficiency of different vessel types or fleets would also help formulate fishery policies, reduce overcapacity and realize the sustainable development of resources^13^. The data envelopment analysis DEA of Danish trawl fleet showed that the efficiency of the fleet was moderate and the fleet operated adequately^14^. The data envelopment analysis of Mediterranean purse seine fleet showed that to achieve sustainable development, it was necessary to reduce the fleet size^15^. The research on purse seine fishing vessels along Aegean coast in Turkey showed that the evaluation of fishing vessels, including fishing methods and fishing gear, was helpful to formulate management policies^16^.

At the same time, technical efficiency, economic efficiency and production efficiency will also impact on fisheries. Using DEA to analyze the fishing fleet, it showed that "captain effect" existed in ports with low efficiency^17^. DEA-Malmquist method was used to analyze the fisheries in China's coastal areas, it showed that the low technical efficiency affected the development of fishing and needed to be further improved^18^.

To evaluate the fishing capacity of fishing vessels, we must first determine the relevant factors that affect the fishing capacity. For this reason, Fang Shuimei and others used DEA to analyze the fishing capacity of gill-netting and net-stretching in Fujian Province, determined the related factors affecting fishing capacity under different operation modes^19,20^. Tao Yajin and others used the standardized CPUE method to analyze the fishing capacity of nine types of fishing vessels in three provinces of South China Sea in 2016, they obtained the ranking of fishing capacity of fishing vessels under different types of fishing vessels^21^. Peter Ward and others made quantitative analysis and research on 11 variables that affect the fishing capacity of ocean fishing tackle vessels, they found that the fishing capacity of big-eye tuna decreased because of the excess fishing capacity, and put forward the reasons for the excess fishing capacity^22^. When applying DEA to obtain indicators, it depends on the reliability of data. At the same time, the more independent variables are input, the more accurate the evaluation results are. Chen Wenhe and others analyzed and studied trawlers in Guangxi Beihai waters by factor analysis method, they found that the four indexes with the most significant influence were fishing vessel operation ability, comprehensive fishing technology, fishing vessel aging degree and sailing rate^23^. Rao Xin and others studied and analyzed the fishing situation in three sea areas of China with the number, total power and total ton of marine fishing vessels as constant input^24^. Damalas D and others took Greek offshore trawlers as the research object, they analyzed the fishing capacity of the fleet with "fishing days", "total power" and "total tonnage" as independent variables^25^.

Fishing vessel fishing capacity evaluation involves many factors, and its evaluation problem is complex multi-attribute analysis and decision-making problems. For complex decision-making problems, decision information is fuzzy and random^26,27^. At present, the primary evaluation methods are analytic hierarchy process, set pair analysis method and fuzzy comprehensive evaluation method. Analytical hierarchy process (AHP) is a classic multi-objective decision-making method, which is widely used, such as evaluating landslide disaster, landslide risk and noise^28–30^. Fuzzy comprehensive evaluation method is based on fuzzy mathematics theory, which is also widely used, such as the evaluation of fishing vessel safety, the evaluation of water resources carrying capacity and the evaluation of parts reliability^31–33^. However, AHP often depends on scoring results, which is subjective and inaccurate for some problems. Although fuzzy comprehensive evaluation method can solve the problem of fuzziness, it has some limitations in dealing with randomness.

Based on fuzzy mathematics and probability statistics, Li Deyi and others put forward a cloud model that can realize qualitative and quantitative interchange, which can better deal with fuzziness and randomness^34^. Based on uncertainty theory, cloud model is widely used in intelligent control, data mining, multi-attribute decision-making and analysis and evaluation. Based on the theory of cloud model, a cloud center of gravity evaluation method was proposed, which used weighted deviation degree to measure the change degree of cloud center of gravity and activated the cloud generator to obtain the evaluation results^35^. Combining the cloud model theory with other methods, it was improved, the measurement and algorithm of cloud model similarity were proposed. At the same time, the application scope was extended to other forms of data, such as S-type cloud model, asymmetric trapezoidal cloud model and Z-trapezoidal conventional cloud model^36–40^. The cloud model was applied to the power industry to evaluate the power development and load response of distribution network, and the obtained results were used to help decision-makers make decisions^41–43^.

The cloud model was combined with AHP weight to evaluate the stability of rock slope, and compared with fuzzy comprehensive evaluation method, it showed that this method was feasible^44^. The cloud model was combined with the AHP to evaluate the degree of soil wind erosion, the uncertainty and fuzziness of AHP were eliminated by using the cloud model, which made the results more convincing^45^. In addition, more objective weights could be obtained by combining entropy weight method with AHP, and more accurate evaluation models could be obtained by blending cloud model theory^46,47^. Cloud model was widely used in multi-condition assessment problems such as energy sustainability assessment, system efficiency assessment and risk assessment^40,49,50^ because of its good handling of randomness and fuzziness.

At present, there is little research on the quantitative evaluation of fishing capacity of single vessel, which covers uncertain multi-factor indicators. However, the evaluation of fishing capacity of single vessel is of great significance to control its fishing capacity and intensity. The traditional evaluation results of fishing capacity of fishing vessels include qualitative and quantitative parts. Qualitative indicators are described in natural language, which is uncertain. Therefore, qualitative concepts should be transformed into quantitative expressions, so that qualitative and quantitative results can be unified and quantified, and the evaluation results can be reflected more reliably.

Based on the cloud model, this paper aims to improve the evaluation index system, apply the cloud model theory, realize the conversion between qualitative and quantitative, and put forward a fishing capacity evaluation method. The evaluation cloud model is obtained by cloud transformation, the cloud rule generator is constructed to make uncertain cloud reasoning, and the cloud drop distribution of fishing capacity of fishing vessels is obtained. The cloud drop distribution reflects the evaluation results of fishing capacity, and the weights of different indexes are considered when making cloud rule reasoning, which makes the evaluation results more reliable and objective.

## Literature and research structure

### Literature

From the previous summary, it can be seen that at present, the main research methods for fishing capacity of fishing vessels are DEA, stochastic frontier method and regression analysis, etc. The main research direction are fishery policy-making^5,6^; surplus fishing capacity^7–9^; the fishing efficiency of fishing vessels and fleets^11–16^; the influence of technical and economic efficiency on fishing capacity^17,18^ and related factors affecting fishing capacity^19–25^ etc. At the same time, for the multi-condition evaluation problem, fuzzy comprehensive evaluation method and analytic hierarchy process are mainly used at present, which are difficult to eliminate or reduce the influence of fuzziness and subjectivity on the evaluation results. The above literature is summarized in Table 1.

**Table 1:** literature summary.

Category	Literature	Main methods and brief content
Fishery policy and excess fishing capacity	Grosskopf^5^ Collier^6^ Pham^7^ Castilla-Espino^8^ Quynh^9^ Ji^10^	DEA method is used to analyze fishery production capacity and fishermen, etc., so as to obtain the degree and reasons of excess fishing capacity, and formulate relevant management policies
Fishing vessels and fleet different ship type fishing efficiency	Gómez^11^ Liang^12^ Quijano^13^ Van Hoof^14^ Tsitsika^15^ Tunca^16^	DEA method is used to analyze fisheries, fishing vessels and fleets in different areas, and the factors affecting fishing efficiency and the influence of different ship types and nets on fishing efficiency are determined
Technical efficiency and production efficiency	Vazquez-RoweI^17^ Li^18^	Using DEA method to analyze the fisheries of different fleets and regions, it is found that the "captain effect" will really affect the fishing efficiency, and the lower technical efficiency will affect the development of fisheries
Factors and indicators affecting fishing capacity	Fang^19^ Fang^20^ Yajin^21^ Ward^22^ Chen^23^ Xin^24^ Damalas^25^	By using DEA, standardized unit fishing effort catch(CPUE) and factor analysis, the fishing capacity of fishing vessels in different countries' sea areas and different types of operations was analyzed, and the relevant factors and indicators affecting fishing capacity were obtained
Analytic hierarchy process, fuzzy comprehensive evaluation method	Panchal^28^ Kim^29^ Li^30^ Wu^31^ Wang^32^ Chen^33^	Analytic hierarchy process (AHP) and fuzzy comprehensive evaluation method are classical evaluation methods, which can be used to evaluate the safety, disaster risk and reliability of parts
Cloud model and its related improvement methods	Yang^35^ Wang^36^ Xie^37^ Li^38^ Wang^39^ Hou^40^ Chen^44^ Guo^45^ Lü^46^ Tan^47^	Combining cloud theory with other methods, the S-shaped cloud model and Z- trapezoidal cloud model are derived. Combining with AHP and entropy weight method, the accuracy of cloud model evaluation is improved and applied in different fields
Application of cloud model evaluation	Zhao^41^ Song^42^ Du^43^ Wang^49^ Hou^50^ Wu^51^	Cloud model and related derivative methods are used to evaluate the development of power industry, power grid security and other aspects, and provide help for management. At the same time, this method is also applied to system efficiency evaluation and sustainability evaluation

Through the above analysis, most of the existing studies use DEA or regression methods to analyze the fishing efficiency and technical efficiency of fishing vessels or fleets, then help to formulate relevant policies. There is a lack of study on the strength evaluation of single-vessel fishing capacity, the relevant indicators used in the research are not comprehensive enough and the evaluation of single-vessel fishing capacity is a multi-condition evaluation problem. Therefore, this study combines previous studies with fishermen's experience to formulate a perfect evaluation index system of single-vessel fishing capacity. Using cloud model theory, combined with AHP and entropy weight method, the qualitative evaluation is transformed into quantitative expression, and the evaluation method of single ship fishing capacity is put forward. Comparing this method with cloud gravity center evaluation method and fuzzy comprehensive evaluation method, the feasibility of this method is verified, and the advantages of this method are shown.

### Research structure

**Figure 1:**
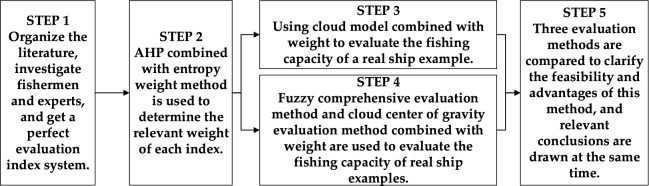
Method flow chart of this paper.

First of all, we should use the summary of past literature and the investigation of fishers and experts to improve the evaluation index system. Secondly, the cloud model theory combined with AHP and entropy weight method is used to form an evaluation method based on cloud model, and a single ship example is evaluated. Thirdly, fuzzy comprehensive evaluation method and cloud center of gravity evaluation method are used to evaluate the fishing capacity of the example. Finally, compare the three methods, and clarify the advantages and rationality. The specific method flow is shown in Fig. 1 below.

## Evaluation index and weight calculation

### Evaluation index system

**Figure 2:**
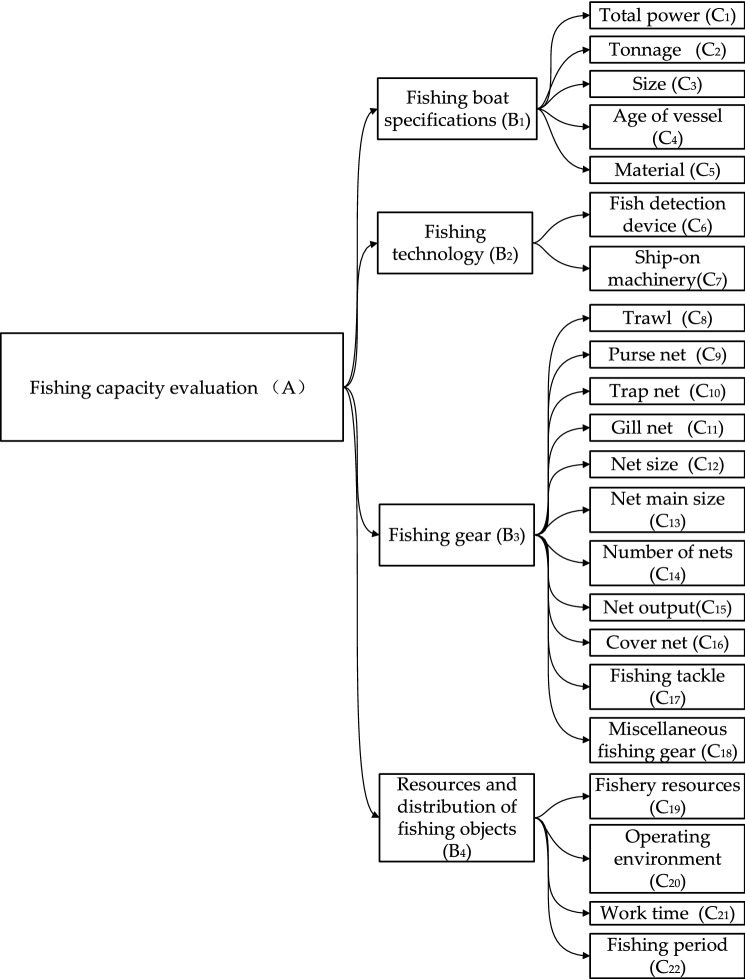
Fishing capacity evaluation index.

The factors that have great influence on fishing capacity are total tonnage, total power, captain and fishing time^23–25^,Based on the investigation results of experts and fishermen, four first-level evaluation indexes are determined as "fishing vessel specifications", "fishing technology", "net gear" and "resources and distribution of fishing objects". The first-level index contains 22 s-level indexes. The specific evaluation index system is shown in Fig. 2 below.

### Index weight calculation

The traditional analytic hierarchy process (AHP) is easily interfered by subjective factors, which makes the evaluation result deviate^22^, so it can be regarded as subjective weight. Entropy method can determine an objective weight according to the fluctuation of data, to reduce the error caused by subjective factors^23^. The weight obtained by entropy method can be regarded as objective weight. Combining the two weights, the obtained weights are more reasonable, and both subjective and objective are taken into account.

#### Calculation subjective index weight

AHP can combine quantitative analysis with qualitative analysis, and establishes an orderly hierarchical structure, compares the weights of the upper elements of two layers, and comprehensively calculates the weights of the lower elements^51,52^.The specific steps are as follows^53^:

- *Step 1*: The objectives of the decision, the factors to be considered (decision criteria) and the decision options are stratified according to their interrelationship.
- *Step 2*: Construct a judgment matrix and represent the elements in the judgment matrix A = (a_*ij*_)_*n*×*n*_ using a 9-bit scale.
- *Step 3*: Single-level sorting and its consistency test. The maximum characteristic root of the matrix A = (a_*ij*_)_*n*×*n*_ is *λ*_*max*_, and the characteristic vector of *λ*_*max*_ is marked as *ω*_*i*_ after normalization, which is the subjective weight vector. The consistency test can be performed according to formula (1).
  1 $$CI = \frac{{\lambda_{\max } - n}}{n - 1} \,RI = \frac{{CI_{1} + CI_{2} + \ldots + CI_{n} }}{n}\,CR = \frac{CI}{{RI}}$$
  where CI is the consistency index, RI is the random consistency index and CR is the consistency ratio.When CR < 0.1, through consistency check, *ω*_*i*_ can be used as a weight vector, otherwise, matrix A is reconstructed.

#### Calculation of objective index weight

The basic idea of entropy weight method is to determine the objective weight according to the variability of indexes.Entropy can not only reflect the degree of information confusion, but also measure the amount of information^53^.If the information entropy of an index is smaller, it indicates that the index is worth more variation, the more information it provides, the greater its role in comprehensive evaluation and the greater its weight.Specifically calculated by the following formulas.

2 $$p_{ij} = \frac{{z_{ij} }}{{\sum\nolimits_{i = 1}^{n} {z_{ij} } }}\, z_{ij} = \frac{{x_{ij} - x_{\min } }}{{x_{\max } - x_{\min } }}$$

3 $$H_{j} = - \frac{1}{\ln n}\sum\nolimits_{i = 1}^{n} {p_{ij} \ln } p_{ij}\,\left( {\sum\nolimits_{j = 1}^{n} {H_{j} = 1,\quad 0 \le H_{j} \le 1} } \right)$$

4 $$\omega_{j} = \frac{{1 - H_{j} }}{{\sum\nolimits_{i = 1}^{n} {1 - H_{j} } }}\;\;\;\;\left( {\sum\nolimits_{j = 1}^{n} {\omega_{j} = 1\;,\;0 \le \omega_{j} \le 1} } \right)\;$$

where *x*_*ij*_ is the membership degree of the i-th object to the j-th index, *x*_*max*_ and *x*_*min*_ are the maximum and minimum values of the index respectively, *P*_*ij*_ is the entropy information of the whole sample, *H*_*j*_ is the entropy value, and *ω*_*j*_ is the entropy weight.

#### Calculation of comprehensive index weight

Comprehensive weight *ω* can be determined by formula (5). Formula (5) combines subjective weight *ω*_*i*_ with objective weight *ω*_*j*_, and *α* and *β* are the comprehensive proportions of subjective weight and objective weight in comprehensive weight^48^.

5 $$\omega = \alpha \omega_{i} + \beta \omega_{j}$$

where *α* and *β* should satisfy *α* + *β* = 1, *α* = 0.7 and *β* = 0.3 can be selected for this method.

## Evaluation methodology

### Cloud model theory

#### Cloud theory related concepts

Cloud model is a model based on probability theory and fuzzy set theory, which transforms the qualitative concept into its quantitative representation through a specially constructed algorithm^54^.In the comment set *X* = {*x*}, the elements in it can map the comment set *x* to another ordered comment set *X'* according to a certain rule *f*. If there is only *X'* corresponding to x in *x'*, then *x'* is the basic variable, and the distribution of membership *μ* in *X'* is called membership cloud^55^.

**Figure 3:**
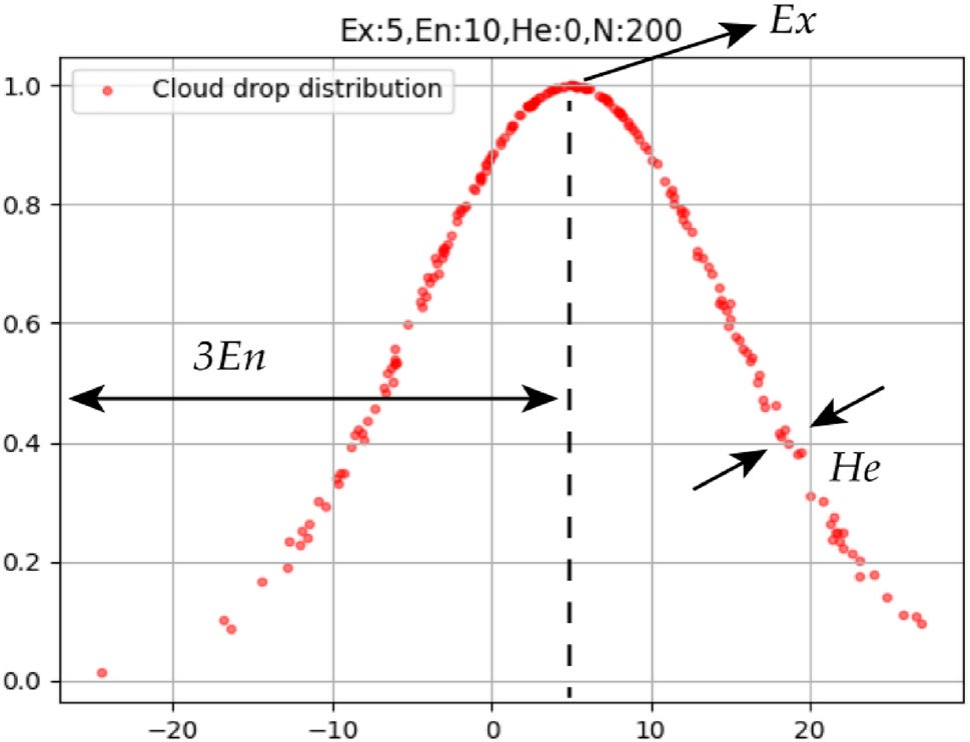
One-dimensional forward cloud cloud drop distribution.

The numerical characteristics of cloud are represented by *Ex*, *En* and *He*, *Ex* is the numerical value that best represents this qualitative concept in the comment set, *En* reflects the range that can be accepted by the concept in the comment set, *He* is the measure of entropy uncertainty, and reflects the randomness of samples of qualitative concept values. The three numerical features are represented in the cloud diagram as shown in Fig. 3.

#### Cloud generator

**Figure 4:**
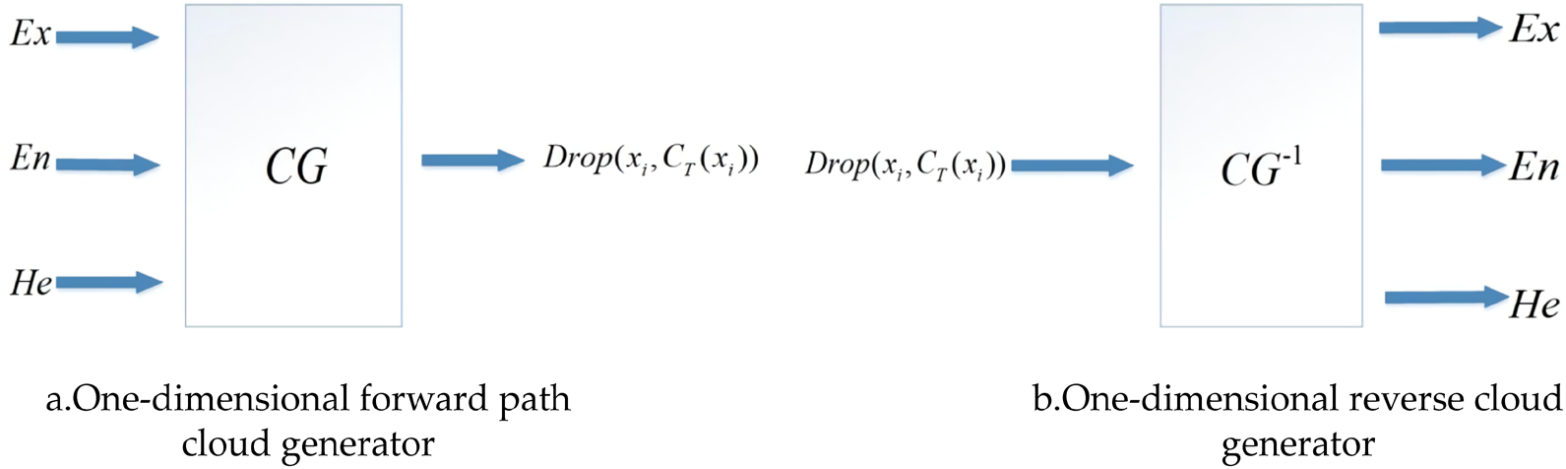
Cloud generator.

The forward path cloud generator is shown in Fig. 4a below. Normal cloud model can reflect the fuzziness and randomness of things or people's cognition in the objective world, and form a mapping between qualitative concepts and quantitative representations^54–56^.

The reverse cloud generator is shown in Fig. 4b below, the function of the reverse cloud generator is to find out the digital features *Ex*, *En* and *He* of the forward cloud generator according to the given cloud droplets, and to convert the quantitative representation into a qualitative concept.

#### Cloud rule generator

**Figure 5:**
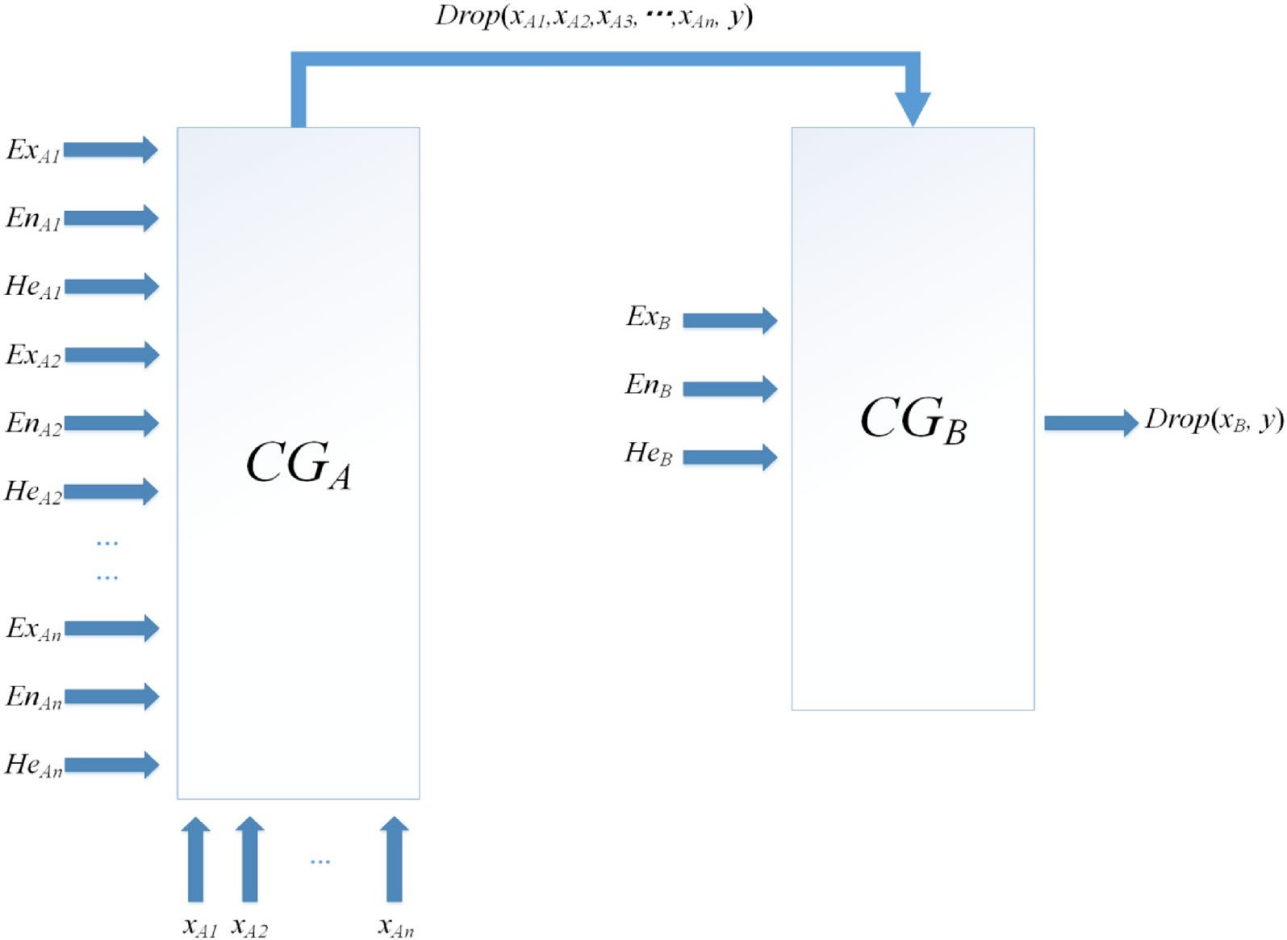
Many conditional single regulations cloud generator.

Cloud rule generator is a tool for uncertainty reasoning. Given the input, after activating the corresponding rule, it outputs the result. The cloud rule generator is composed of a preceding cloud generator and a succeeding cloud generator. The schematic diagram of the cloud rule generator is shown in Fig. 5.

The rules for uncertainty reasoning are as follows^56^: if A_1_, A_2_, …, A_n_ then B.

### Cloud model evaluation method

The evaluation method based on cloud model includes the following steps:

- Step 1: Building an evaluation index system and calculating relevant weights(The weight solution has been introduced in the third part);
- Step 2: Generate a standard comment cloud model;
- Step 3: Acquire expert scores and generate an expert score cloud model;
- Step 4: Use the cloud reasoning system for reasoning to obtain the evaluation result.

#### Generate standard comment cloud model

The evaluation index comment set is set as *C* = {*C*_*1*_*, C*_*2*_*, C*_*3*_*, C*_*4*_},and the corresponding score interval is:Excellent (90 ~ 100), Good (80 ~ 90), Medium (60 ~ 80) and Poor (0 ~ 60), Among them, "Excellent" and "Poor" are unilateral constraint comments, while "Good" and "Medium" are bilateral constraint comments, which can be described by one-dimensional normal forward cloud model.The digital characteristics of the forward cloud generator can be calculated by formulas (6)–(7).

6 $$\left\{ \begin{gathered} Ex = X_{ + def} \;or\;X_{ - def} \hfill \\ En = {{(X_{ + def} - X_{\min } )} \mathord{\left/ {\vphantom {{(X_{ + def} - X_{\min } )} {3\;or\;{{(X_{\max } - X_{ - def} )} \mathord{\left/ {\vphantom {{(X_{\max } - X_{ - def} )} 3}} \right. \kern-\nulldelimiterspace} 3}}}} \right. \kern-\nulldelimiterspace} {3\;or\;{{(X_{\max } - X_{ - def} )} \mathord{\left/ {\vphantom {{(X_{\max } - X_{ - def} )} 3}} \right. \kern-\nulldelimiterspace} 3}}} \hfill \\ He = k \hfill \\ \end{gathered} \right.$$

7 $$\left\{ \begin{gathered} Ex = {{(X_{\min } + X_{\max } )} \mathord{\left/ {\vphantom {{(X_{\min } + X_{\max } )} 2}} \right. \kern-\nulldelimiterspace} 2} \hfill \\ En = {{(X_{\max } - X_{\min } )} \mathord{\left/ {\vphantom {{(X_{\max } - X_{\min } )} 6}} \right. \kern-\nulldelimiterspace} 6} \hfill \\ He = k \hfill \\ \end{gathered} \right.$$

In formula (6), the value range of constraint comments is [*X*_*-def*_, *X*_*max*_] or [*X*_*min*_, *X*_+*def*_], which is a unilateral constraint comment.In formula (7), the value range of constraint comments is [*X*_*min*_, *X*_*max*_], which is bilateral constraint comments. According to experience, the value of *k* can be *En/10*, and the specific value should be combined with the actual situation.

#### Comprehensive cloud model for obtain expert comments

A rating range is used as input to the inverse cloud generator instead of the determined rating values, and the data features of the combined expert review cloud model are computed using each of the generated expert review cloud models^58^. Formula (8) can be used to calculate the comprehensive cloud numerical characteristics of *n* expert scores.

8 $$\left\{ \begin{gathered} Ex = {{\left( {Ex_{1} En_{1} + Ex_{2} En_{2} + \ldots + Ex_{n} En_{n} } \right)} \mathord{\left/ {\vphantom {{\left( {Ex_{1} En_{1} + Ex_{2} En_{2} + \ldots + Ex_{n} En_{n} } \right)} {\left( {En_{1} + En_{2} + \ldots + En_{n} } \right)}}} \right. \kern-\nulldelimiterspace} {\left( {En_{1} + En_{2} + \ldots + En_{n} } \right)}} \hfill \\ En = En_{1} + En_{2} + \ldots + En_{n} \hfill \\ He = {{(He_{1} En_{1} + He_{2} En_{2} + \ldots He_{n} En_{n} )} \mathord{\left/ {\vphantom {{(He_{1} En_{1} + He_{2} En_{2} + \ldots He_{n} En_{n} )} {(En_{1} + En_{2} + \ldots + En_{n} )}}} \right. \kern-\nulldelimiterspace} {(En_{1} + En_{2} + \ldots + En_{n} )}} \hfill \\ \end{gathered} \right.\;$$

where the evaluation cloud features of n expert scores are (*Ex*_*1*_, *Ex*_*2*_, …, *Ex*_*n*_), (*En*_*1*_, *En*_*2*_, …, *En*_*n*_) and (*He*_*1*_, *He*_*2*_, …, *He*_*n*_).

#### Building a cloud reasoning system

**Figure 6:**
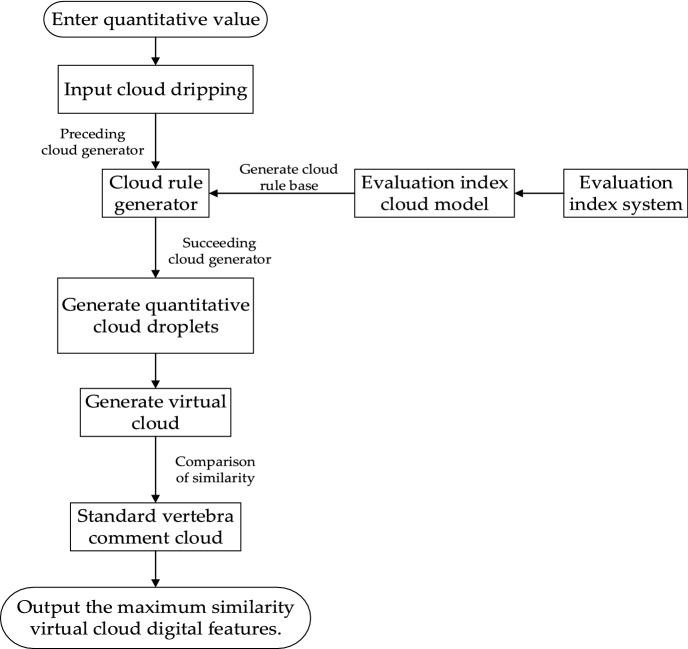
Cloud reasoning system workflow.

The cloud reasoning system consists of a cloud rule generator and a cloud rule base. After the system gets the input, a virtual cloud is generated by the cloud rule generator, and the virtual cloud is compared with the standard cloud model, and the comment corresponding to the cloud model with the highest similarity is the evaluation result. The specific workflow is shown in Fig. 6 below.

The construction steps of the cloud reasoning system are as follows:

- *Step 1*: Build a cloud rule base and enter any value to activate the corresponding rule.
- *Step 2*: A cloud computing rule generator is constructed, the weights are combined with an algorithm to decompose the multi-conditional cloud computing rule generator into several one-dimensional generators, and the output value of each one-dimensional cloud computing generator is calculated, and the weighted average method is used to obtain the final output^57^.
- *Step 3*: Calculate the cloud graph similarity as a way to compare the similarity between the virtual cloud and the standard cloud model^59,60^.
  The definition of similarity is as follows: Let two cloud images *C*_*1*_(*Ex*_*1*_, *En*_*1*_, *He*_*1*_) and *C*_*2*_(*Ex*_*2*_, *En*_*2*_, *He*_*2*_). If the membership degree of cloud drops (*x*_*i*_, *μ*_*i*_) generated by *C*_*1*_ inverse cloud generator in *C*_*2*_ cloud is *μ*_*i*_^*’*^, then the similarity between clouds *C*_*1*_ and *C*_*2*_ is $\frac{1}{n}\sum\limits_{i = 1}^{n} {\mu_{i}^{^{\prime}} }$, which is recorded as *δ*^58^.

### Cloud-gravity-center Assessing

Cloud-gravity-center Assessing uses the change of cloud gravity center to express the change of evaluation information. The change of cloud center of gravity reflects the change of information center, and the cloud center of gravity is expressed by formula (9).

9 $$T = a \times b$$

where *a* is the position of cloud center of gravity and *b* is the height of cloud center of gravity.

The Cloud-gravity-center Assessing is implemented as follows^35^:

Step1: A cloud model is used to represent each indicator whose numerical characteristics are calculated by formulas (10)–(11).

10 $$En = \frac{{x_{1} + x_{2} + \ldots + x_{n} }}{n}$$

11 $$En = \frac{{\max \left( {x_{1} ,\;x_{2} ,\; \ldots ,\;x_{n} } \right) - \min \left( {x_{1} ,\;x_{2} ,\; \ldots ,\;x_{n} } \right)}}{6}$$

where *Ex* and *En* are the expectation and entropy of the cloud model respectively, and *x*_*i*_ is the data of sample.

Step2: When the system changes, the integrated cloud model characterizing the system state also changes, and its center of gravity vector changes from *T* = {*T*_*1*_, *T*_*2*_, …, *T*_*p*_} to *T*^*’*^ = {*T*_*1*_^*’*^, *T*_*2*_^*’*^, …, *T*_*p*_^*’*^}.

Step3: Using the weights of the indicators, the weighted offset is calculated from formulas (12–15).

12 $$T^{0} = a^{0} \times b$$

13 $$T = a \times b$$

14 $$T_{i}^{G} = \left\{ \begin{gathered} {{\left( {T_{i} - T_{i}^{0} } \right)} \mathord{\left/ {\vphantom {{\left( {T_{i} - T_{i}^{0} } \right)} {T_{i}^{0} \quad \quad T_{i} < T_{i}^{0} }}} \right. \kern-\nulldelimiterspace} {T_{i}^{0} \quad \quad T_{i} < T_{i}^{0} }} \hfill \\ {{\left( {T_{i} - T_{i}^{0} } \right)} \mathord{\left/ {\vphantom {{\left( {T_{i} - T_{i}^{0} } \right)} {T_{i} \quad \quad T_{i} \ge T_{i}^{0} }}} \right. \kern-\nulldelimiterspace} {T_{i} \quad \quad T_{i} \ge T_{i}^{0} }} \hfill \\ \end{gathered} \right.$$

15 $$\theta = \sum\nolimits_{j = 1}^{p} {\left( {\omega_{j}^{*} \times T_{i}^{G} } \right)}$$

where *a*^*0*^ is the cloud center of gravity position vector in the ideal state, *a* is the cloud center of gravity position vector in the current state, *b* is the cloud center of gravity height, *T*_*i*_^*G*^ is the normalized vector of *T*,*T*^*0*^ is the cloud barycenter vector*, ω*_j_^*^ is the weight of each index, and *θ* is the weighted deviation degree.

### Fuzzy comprehensive evaluation method

The fuzzy comprehensive evaluation method converts qualitative evaluation into quantitative evaluation using the theory of affiliation, and the evaluation results are determined by the principle of maximum affiliation.

The judgment matrix B and the final evaluation weight A are obtained according to the affiliation relationship, and the comment corresponding to the maximum value in vector B is the final evaluation result, which can be calculated by formulas (16)–(17)^32^.

16 $$R = \left\{ {\begin{array}{*{20}c} {r_{11} } & {r_{12} } & \cdots & {r_{1n} } \\ {r_{21} } & {r_{22} } & \cdots & {r_{2n} } \\ \vdots & \vdots & \ddots & \vdots \\ {r_{m1} } & {r_{m2} } & \cdots & {r_{mn} } \\ \end{array} } \right\}$$

17 $$B = A \times R$$

where the matrix R consists of the single-factor evaluation set single-factor evaluation set *r*_*i*_ = {*r*_*i1*_, *r*_*i2*_, …, *r*_*in*_}, A = {*a*_*1*_, *a*_*2*_, …, *a*_*m*_} is the weight vector, *a*_*i*_ is the weight of each factor, and B is the judgment matrix.

## Case analysis

Take the single trawler * Yangjiang Fishing 0**8 as an example to evaluate the fishing capacity, and the comment set is *V* = {*V*_*1*_*,V*_*2*_*,V*_*3*_*,V*_*4*_} = {Excellent, Good, Medium, Poor}. The specific information of the evaluation indicators is shown in Table 2 below.

**Table 2:** Fishing vessels indicator information.

First indicator	Secondary indicator	Parameter
Specification	Size/m	37.80
	Total power/kW	305
	Tonnage/t	330
	Age/year	5
	Material	Steel
Net	Trawl	Polyethylene single trawl
	Number of nets	1
	Net output/ kg	50
	Net main size	**8% lower than the average trawl net size in the operation area
	Net size	**44 mm
Fishing technology	Ship-on machinery	Mechanization, low degree of automation and frequent human intervention
	Fish detection device	Fish detection instrument: FS1001B
Resources and distribution of fishing objects	Fishery resources	***Fishing ground Coordinate: (1***.23, 1*.54)
	Fishing period	Start: 2019-12-24 08:20:00 End: 2019-12-24 14:06:00
	Operating environment	The working environment is moderate and the wind speed is 8–13.8 m/s
	Work time/h	6

### Determining index weight

The comparison matrix of each index is shown in the following Tables 3, 4, 5, 6 and 7. The weights of the evaluation index system are also obtained by combining formulas (1)–(5) with expert scoring, as shown in Table 8 below.

**Table 3:** Comparative criteria level matrix (Level 1 indicators).

A_ij_	B_1_	B_2_	B_3_	B_4_
B_1_	1	3	2	3
B_2_	1/3	1	1/2	1
B_3_	1/2	2	1	2
B_4_	1/3	1	1/2	1

**Table 4:** Comparative criteria level matrix (Level 2 indicators).

A_ij_	C_1_	C_2_	C_3_	C_4_	C_5_
C_1_	1	2	2	4	3
C_2_	1/2	1	2	3	2
C_3_	1/2	1/2	1	4	4
C_4_	1/4	1/3	1/4	1	1/3
C_5_	1/3	1/2	1/4	3	1

**Table 5:** Comparative criteria level matrix (Level 2 indicators).

A_ij_	C_6_	C_7_
C_6_	1	1/2
C_7_	2	1

**Table 6:** Comparative criteria level matrix (Level 2 indicators).

A_ij_	C_8_	C_9_	C_10_	C_11_	C_12_	C_13_	C_14_	C_15_	C_16_	C_17_	C_18_
C_8_	1	2	3	3	4	4	5	5	5	6	6
C_9_	1/2	1	2	2	2	2	4	4	4	5	5
C_10_	1/3	1/2	1	1	2	2	3	3	3	4	4
C_11_	1/3	1/2	1	1	2	2	3	3	3	4	4
C_12_	1/4	1/2	1/2	1/2	1	1	2	2	2	3	3
C_13_	1/4	1/2	1/2	1/2	1	1	2	2	2	2	3
C_14_	1/5	1/4	1/3	1/3	1/2	1/2	1	1	1	2	2
C_15_	1/5	1/4	1/3	1/3	1/2	1/2	1	1	1	2	2
C_16_	1/5	1/4	1/3	1/3	1/2	1/2	1	1	1	2	2
C_17_	1/6	1/5	1/4	1/4	1/3	1/2	1/2	1/2	1/2	1	2
C_18_	1/6	1/5	1/4	1/4	1/3	1/3	1/2	1/2	1/2	1/2	1

**Table 7:** Comparative criteria level matrix (Level 2 indicators).

A_ij_	C_19_	C_20_	C_21_	C_22_
C_19_	1	3	3	2
C_20_	1/3	1	2	1/2
C_21_	1/3	1/2	1	1/3
C_22_	1/2	2	3	1

**Table 8:** Evaluation index and its weight.

First indicator	AHP	Entropy weight method	Consolidated weight	Secondary indicator	AHP	Entropy weight method	Consolidated weight
Specification	0.46	0.137	0.36310	Size	0.230	0.0534	0.17702
				Total power	0.354	0.0696	0.26868
				Tonnage	0.243	0.0860	0.19590
				Age	0.061	0.3887	0.15931
				Material	0.112	0.4023	0.19909
Net	0.26	0.182	0.23660	Number of nets	0.044	0.1002	0.06086
				Trawl	0.258	0.0317	0.19011
				Gill net	0.118	0.0399	0.09457
				Trap net	0.118	0.0723	0.10429
				Purse net	0.168	0.0417	0.13011
				Fishing tackle	0.031	0.1176	0.05698
				Cover net	0.044	0.1140	0.06500
				Miscellaneous fishing gear	0.026	0.1408	0.06044
				Net main size	0.073	0.0944	0.07942
				Net size	0.076	0.1251	0.09073
				Net output	0.044	0.1223	0.06749
Fishing technology	0.14	0.2427	0.17081	Fish detection device	0.33	0.545	0.3945
				Ship-on machinery	0.67	0.455	0.6055
Resources and distribution of fishing objects	0.14	0.4383	0.22949	Fishery resources	0.45	0.0560	0.33180
				Fishing period	0.28	0.1191	0.23173
				Operating environment	0.16	0.3613	0.22039
				Work time	0.11	0.4636	0.21608

### Build standard cloud model and scoring cloud model

**Figure 7:**
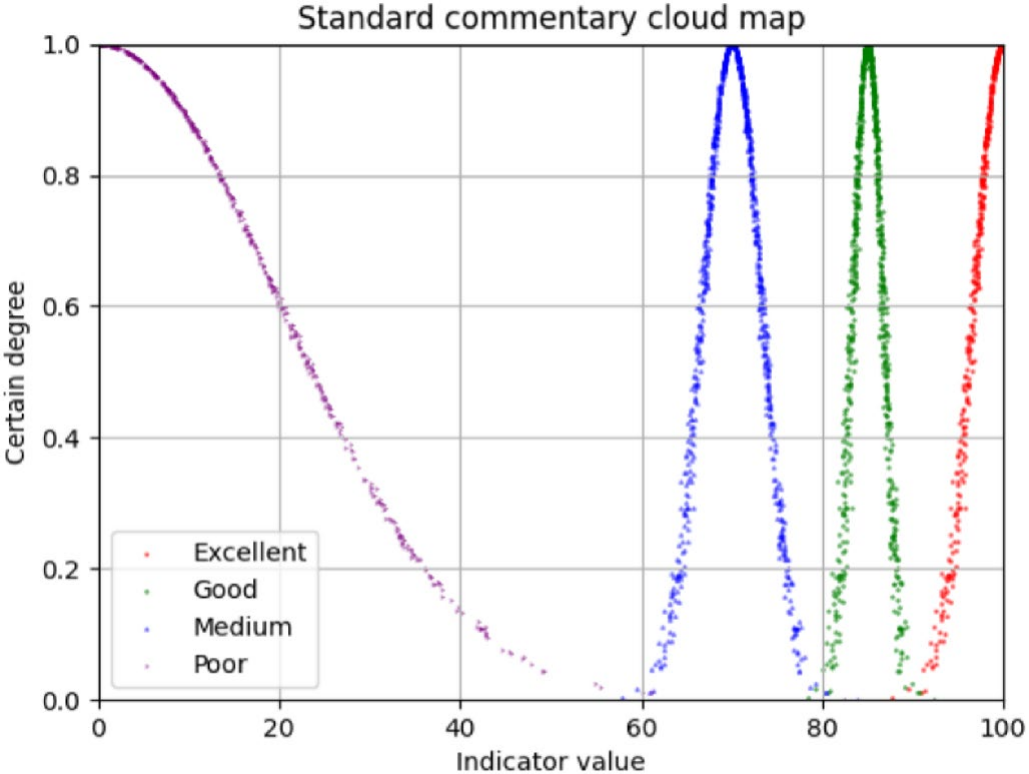
Standard comments volume cloud.

The numerical characteristic of that standard comment cloud model can be calculated by formulas (6)–(7), and the results are shown in Table 9, and the standard comment cloud model is shown in Fig. 7.

**Table 9:** Standard Review Cloud Model.

Evaluation grade	Excellent	Good	Medium	Poor
Score interval	(90–100)	(80–90)	(60–80)	(0–60)
Digital feature of evaluation	(100,3.3,0.2)	(85,1.7,0.2)	(70,3.3,0.2)	(0,20,0.2)

Ten experts and fishermen scored the indicators, and the scoring results are shown in Tables 10, 11, 12 and 13. Since the selected calculation example is a single trawler, the indicators of gill net, gear, seine, fishing industry, net cover, and miscellaneous fishing gear do not need to be scored and the cloud model is (0,0,0), and the comprehensive evaluation cloud model of experts for each indicator is calculated by formula (8), and the results are shown in Tables 14, 15, 16 and 17 below.

**Table 10:** Scoring result (specification).

Scoring result\indicator	Size	Total power	Tonnage	Age	Material
Expert 1	73–85	78–82	80–90	90–100	84–94
Expert 2	80–90	80–90	65–75	50–55	80–90
Expert 3	75–85	69–79	57–64	78–88	65–78
Expert 4	90–92	67–69	87–92	96–98	30–42
Expert 5	91–95	61–67	76–83	65–75	82–89
Expert 6	63–79	66–69	60–70	90–100	45–55
Expert 7	79–91	80–92	88–100	71–85	80–90
Expert 8	73–84	80–90	91–95	90–95	81–90
Expert 9	80–90	79–80	80–91	59–62	85–93
Expert 10	80–85	80–85	80–85	80–91	75–80

**Table 11:** Scoring result(Net).

Scoring result\indicator	Trawl	Number of nets	Net output	Net main size	Net size
Expert 1	85–94	85–97	82–96	90–94	91–96
Expert 2	90–100	80–90	80–90	90–100	90–100
Expert 3	60–65	50–55	70–75	40–45	50–55
Expert 4	68–85	95–98	77–86	95–100	80–88
Expert 5	95–98	59–60	51–54	47–57	51–59
Expert 6	58–68	87–90	83–93	90–98	91–98
Expert 7	90–95	66–77	78–89	78–86	75–89
Expert 8	91–93	87–92	89–95	82–96	86–93
Expert 9	78–93	65–78	74–79	77–87	80–93
Expert 10	70–75	80–91	90–100	70–75	85–95

**Table 12:** Scoring result(Fishing technology).

Scoring result\Indicator	Ship-on machinery	Fish detection device
Expert 1	83–91	80–90
Expert 2	70–80	80–85
Expert 3	75–80	58–70
Expert 4	82–86	92–94
Expert 5	69–79	66–69
Expert 6	97–100	90–100
Expert 7	80–90	80–90
Expert 8	85–91	87–100
Expert 9	78–90	90–95
Expert 10	90–100	79–80

**Table 13:** Scoring result(Resources and distribution of fishing objects).

Scoring result\Indicator	Fishery resources	Fishing period	Operating environment	Work time
Expert 1	77–87	89–96	77–90	81–91
Expert 2	70–80	80–90	79–90	80–90
Expert 3	65–77	45–58	77–83	83–84
Expert 4	91–95	78–85	69–76	83–91
Expert 5	91–99	34–50	90–98	54–69
Expert 6	79–91	83–93	79–89	88–100
Expert 7	89–100	70–80	93–100	70–87
Expert 8	90–100	93–100	60–70	95–100
Expert 9	80–90	70–80	54–61	70–80
Expert 10	90–95	90–95	90–95	90–95

**Table 14:** Fishing vessels specification index expert evaluation cloud model.

Indicator	Size	Total power	Tonnage	Age	Material
Digital feature of evaluation	(80.80,57.66,3.6)	(79.47,39.49,2.7)	(80.00,50.77,2.9)	(82.04,50.13,3.2)	(74.28,60.80,3.3)

**Table 15:** Net tare expert evaluation cloud model.

Indicator	Trawl	Gill net	Trap net	Purse net	Fishing tackle	Cover net	Miscellaneous fishing gear	Number of nets	Net main size	Net size	Net output
Digital feature of evaluation	(83.04,45.11,3.00)	(0,0,0)	(0,0,0)	(0,0,0)	(0,0,0)	(0,0,0)	(0,0,0)	(80.04,46.37,3.20)	(80.69,49.50,2.97)	(83.37,54.52,3.17)	(84.73,52.01,3.13)

**Table 16:** Fishing technical indicator expert evaluation cloud model.

Indicator	Ship-on machinery	Fish detection device
Digital feature of evaluation	(83.86,48.89,2.92)	(84.53,44.50,3.13)

**Table 17:** Resources and distribution indicators of fishing objects Evaluation cloud model.

Indicator	Fishery resources	Fishing period	Operating environment	Work time
Digital feature of evaluation	(85.61,57.66,3.20)	(73.11,59.56,3.44)	(74.64,52.65,2.97)	(81.44,58.28,3.78)

### Cloud reasoning

The cloud rule base is constructed as follows:

Cloud rules are constructed in the form of "if A_1_, A_2_, …, then B". Taking five secondary indicators under "fishing vessel specifications" as examples, the rule base of multi-condition single rules.

The obtained expert evaluation cloud model, combined with Table 9 and comment set, can activate relevant rules, taking fishing vessel specification evaluation as an example.Through the multi-condition single rule algorithm combined with weight, two cloud droplets are obtained as follows: *x*_*1*_ = 84.75220327, *x*_*2*_ = 69.52946857.

According to the inverse cloud generator algorithm, the numerical characteristics of the comprehensive evaluation cloud obtained from two cloud droplets are: (77.1408, 1.6897, 0.0).

According to the cloud image similarity calculation algorithm, the digital characteristics of comprehensive evaluation cloud are compared with those of standard cloud, and the obtained similarity is shown in Table 18 below.

**Table 18:** Fishing vessels specification indicator cloud map similarity.

Evaluation grade	Excellent	Good	Medium	Poor
Standard review cloud model	(100,3.3,0.2)	(85,1.7,0.2)	(70,3.3,0.2)	(0,20,0.2)
Similarity	0.0178	0.0430	0.1074	0.0056

**Figure 8:**
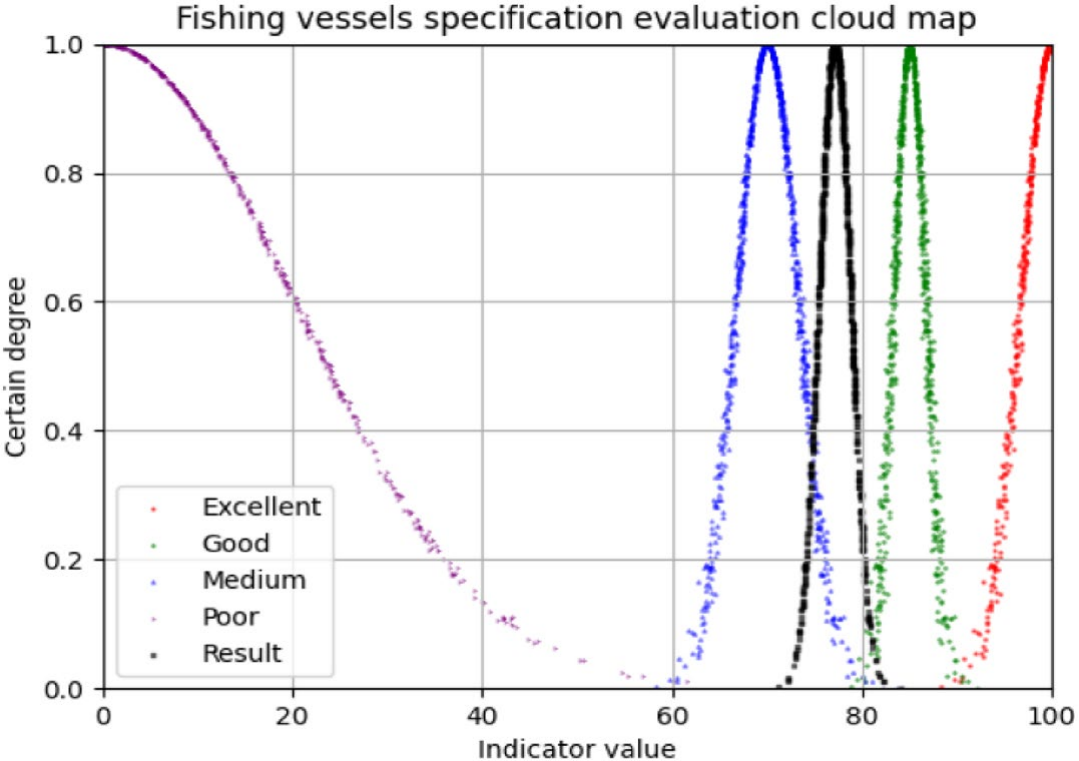
Fishing vessels specification evaluation cloud map.

According to Table 18, the virtual cloud should be between "Medium" and "Good", and the result is partial to "Medium". Through the forward cloud generator, the comprehensive evaluation cloud is drawn on the standard cloud-scale, and the evaluation cloud picture of fishing vessel specifications is shown in Fig. 8 below.

As can be seen from Fig. 8, the evaluation cloud picture is between the standard evaluation values of "Medium" and "Good", so the similarity with "Good" and "Medium" is higher than the other two evaluations. Among the two evaluations of "Medium" and "Good", the highest similarity is taken as the final evaluation of fishing vessel specifications, that is, the fishing vessel specifications are "Medium". When this method is used for evaluation, the evaluation cloud and the standard cloud map can be generated, and the similarity between the evaluation cloud and the cloud maps on both sides can be directly calculated. The larger similarity is the evaluation result.

### Method comparison and result analysis

Using the scores of experts and fishermen, the fuzzy comprehensive evaluation method and the "cloud-gravity-center" evaluation method were used to evaluate the fishing capacity of fishing boats. Taking the evaluation of fishing vessel specifications as an example, the affiliation vector of fuzzy comprehensive evaluation of fishing vessel specifications was obtained, and the evaluation results were obtained by inverse fuzzification. As shown in Table 19 below, the evaluation result is "medium" according to the principle of maximum membership. The weighted deviation *θ* = -0.2081 is obtained by "cloud-gravity-centered evaluation", and 0.7919 is input into the cloud generator, and the final activation result is "medium". The comparison results of the three methods are shown in Table 20 below, and the results of the three evaluation methods are the same, which verifies the feasibility of the method.

**Table 19:** The result of Fuzzy comprehensive evaluation method.

Evaluation grade	Excellent	Good	Medium	Poor
Degree of membership	0.158217	0.388047	0.397987	0.055749
Evaluation results	76.664785

**Table 20:** Evaluation results comparison.

Evaluation method	Cloud model	Fuzzy comprehensive evaluation method	Cloud evaluation center of gravity
Evaluation results	(77.1408, 1.6897, 0.0)	76.664785	0.7919

The comparison from Table 20 reveals that the results obtained from the cloud model contain three numerical features that can reflect the evaluation results more comprehensively, where "*Ex*" reflects the average level of the evaluation results, i.e., the current level of the evaluation object, and "*En*" reflects the dispersion of the cloud image, i.e., the reliability of the current evaluation results." The larger "*En*" is, the lower the reliability of the results, while "*He*" reflects the condition of the cloud drops^58^, and the evaluation results are described by three numerical features together, which improves the persuasiveness of the results. And the results obtained by the cloud computing method can be clearly pictorialized (e.g., Fig. 8), and each point in the image represents the quantitative concept transformed by the qualitative concept, and the accuracy of the conversion is reflected by the degree of certainty(vertical coordinate), and the pictorialized evaluation results make the evaluation results more intuitive. At the same time, the cloud computing method also compares the evaluation results in terms of cloud map similarity, which further improves the accuracy of the results.

Compared with the cloud model calculation method, the fuzzy comprehensive evaluation method can realize the transformation from qualitative to quantitative according to the principle of maximum affiliation, but its calculation process is more complicated, and the evaluation process completely relies on subjective scoring, and the results are often more subjective and fuzzy.

The cloud-gravity-centered evaluation method has a simpler calculation process and can better reduce the influence of subjective scoring on the results, but it only relies on a numerical feature to describe the evaluation results, which may make the results less accurate in practical application due to the one-sidedness and singularity of information.

## Conclusion

Aiming at the problems in the evaluation process of fishing capacity of fishing vessels, such as incomplete evaluation indexes, mixed qualitative and quantitative descriptions, ambiguous indexes and substantial uncertainty, this paper puts forward a quantitative evaluation of fishing capacity of single vessel based on cloud model theory, and evaluates fishing capacity of single vessel by combining four first-class indexes such as fishing vessel specifications, fishing gear, fishing technology and resource distribution and corresponding second-class indexes. The research shows that:

1. The traditional qualitative inspection and scoring evaluation methods cannot accurately describe the evaluation object. In this study, the qualitative description of evaluation can be transformed into quantitative evaluation through scoring interval conversion and cloud model processing, and objective, accurate and unified quantitative evaluation results can be obtained.
2. The three numerical characteristics of the comprehensive evaluation cloud are obtained based on the cloud model theory, and the three numerical characteristics have different meanings, so it is able to consider the fishing vessel fishing capacity evaluation from both the quantitative results and the reliability of the results. For the decision evaluation problem with complex multi-attribute factors, the objectivity and accuracy of the evaluation results can be further improved.
3. Using the same-scale cloud images to directly reflect the evaluation results, the similarity comparison of cloud model provides objective basis for the cloud image results, and the cloud model theory is very suitable for solving the problem of fishing vessel performance evaluation covering multi-attribute uncertain factors. This study promotes the application of uncertainty information theory in the field of fishing vessel performance analysis, mining and evaluation decision engineering.

The evaluation of fishing capacity of fishing vessels depends on a complete and objective evaluation index system and an evaluation method that can minimize subjective influence. Perfecting the index system to describe fishing capacity of fishing vessels more accurately, and proposing a completely quantitative evaluation system that does not depend on subjective scores will be the next research work.

## Supplementary Information

Supplementary Information 1.

Supplementary Information 2.

Supplementary Information 3.

## Data Availability

The data come from the data on offshore fishing operations in China obtained from the China Fishery Statistical Yearbook published from 2004 to 2020.
