# Supplementary material for: Fishing capacity evaluation of fishing vessel based on cloud model
Source: Sci Rep. 2022 May 28;12:8976. doi: 10.1038/s41598-022-12852-8 (PMC9148315; doi:10.1038/s41598-022-12852-8)
Supplement: Supplementary file 1 — Supplementary Information 1. [file 41598_2022_12852_MOESM1_ESM.docx]

Table 1 Fishing vessels indicator information

| First indicator | Secondary indicator | Parameter |
| --- | --- | --- |
| Specification | Size/m | 37.80 |
|  | Total power/kW | 305 |
|  | Tonnage/t | 330 |
|  | Age/year | 5 |
|  | Material | Steel |
| Net | Trawl | Polyethylene single trawl |
|  | Number of nets | 1 |
|  | Net output/ kg | 50 |
|  | Net main size | **8% lower than the average trawl net size in the operation area |
|  | Net size | **44mm |
| Fishing technology | Ship-on machinery | Mechanization, low degree of automation and frequent human intervention |
|  | Fish detection device | Fish detection instrument: FS1001B |
| Resources and distribution of fishing objects | Fishery resources | ***Fishing ground Coordinate: (1***.23，1*.54) |
|  | Fishing period | Start: 2019-12-24 08:20:00  End: 2019-12-24 14:06:00 |
|  | Operating environment | The working environment is moderate and the wind speed is 8~13.8m/s |
|  | Work time/h | 6 |
