# Supplementary material for: Fishing capacity evaluation of fishing vessel based on cloud model
Source: Sci Rep. 2022 May 28;12:8976. doi: 10.1038/s41598-022-12852-8 (PMC9148315; doi:10.1038/s41598-022-12852-8)
Supplement: Supplementary file 2 — Supplementary Information 2. [file 41598_2022_12852_MOESM2_ESM.docx]

Table 1 Scoring result(Specification)

| Scoring result\Indicator | Size | Total power | Tonnage | Age | Material |
| --- | --- | --- | --- | --- | --- |
| Expert 1 | 73~85 | 78~82 | 80~90 | 90~100 | 84~94 |
| Expert 2 | 80~90 | 80~90 | 65~75 | 50~55 | 80~90 |
| Expert 3 | 75~85 | 69~79 | 57~64 | 78~88 | 65~78 |
| Expert 4 | 90~92 | 67~69 | 87~92 | 96~98 | 30~42 |
| Expert 5 | 91~95 | 61~67 | 76~83 | 65~75 | 82~89 |
| Expert 6 | 63~79 | 66~69 | 60~70 | 90~100 | 45~55 |
| Expert 7 | 79~91 | 80~92 | 88~100 | 71~85 | 80~90 |
| Expert 8 | 73~84 | 80~90 | 91~95 | 90~95 | 81~90 |
| Expert 9 | 80~90 | 79~80 | 80~91 | 59~62 | 85~93 |
| Expert 10 | 80~85 | 80~85 | 80~85 | 80~91 | 75~80 |

Table 2 Scoring result(Net)

| Scoring result\Indicator | Trawl | Number of nets | Net output | Net main size | Net size |
| --- | --- | --- | --- | --- | --- |
| Expert 1 | 85~94 | 85~97 | 82~96 | 90~94 | 91~96 |
| Expert 2 | 90~100 | 80~90 | 80~90 | 90~100 | 90~100 |
| Expert 3 | 60~65 | 50~55 | 70~75 | 40~45 | 50~55 |
| Expert 4 | 68~85 | 95~98 | 77~86 | 95~100 | 80~88 |
| Expert 5 | 95~98 | 59~60 | 51~54 | 47~57 | 51~59 |
| Expert 6 | 58~68 | 87~90 | 83~93 | 90~98 | 91~98 |
| Expert 7 | 90~95 | 66~77 | 78~89 | 78~86 | 75~89 |
| Expert 8 | 91~93 | 87~92 | 89~95 | 82~96 | 86~93 |
| Expert 9 | 78~93 | 65~78 | 74~79 | 77~87 | 80~93 |
| Expert 10 | 70~75 | 80~91 | 90~100 | 70~75 | 85~95 |

Table 3 Scoring result(Fishing technology)

| Scoring result\Indicator | Ship-on machinery | Fish detection device |
| --- | --- | --- |
| Expert 1 | 83~91 | 80~90 |
| Expert 2 | 70~80 | 80~85 |
| Expert 3 | 75~80 | 58~70 |
| Expert 4 | 82~86 | 92~94 |
| Expert 5 | 69~79 | 66~69 |
| Expert 6 | 97~100 | 90~100 |
| Expert 7 | 80~90 | 80~90 |
| Expert 8 | 85~91 | 87~100 |
| Expert 9 | 78~90 | 90~95 |
| Expert 10 | 90~100 | 79~80 |

Table 4 Scoring result(Resources and distribution of fishing objects)

| Scoring result\Indicator | Fishery resources | Fishing period | Operating environment | Work time |
| --- | --- | --- | --- | --- |
| Expert 1 | 77~87 | 89~96 | 77~90 | 81~91 |
| Expert 2 | 70~80 | 80~90 | 79~90 | 80~90 |
| Expert 3 | 65~77 | 45~58 | 77~83 | 83~84 |
| Expert 4 | 91~95 | 78~85 | 69~76 | 83~91 |
| Expert 5 | 91~99 | 34~50 | 90~98 | 54~69 |
| Expert 6 | 79~91 | 83~93 | 79~89 | 88~100 |
| Expert 7 | 89~100 | 70~80 | 93~100 | 70~87 |
| Expert 8 | 90~100 | 93~100 | 60~70 | 95~100 |
| Expert 9 | 80~90 | 70~80 | 54~61 | 70~80 |
| Expert 10 | 90~95 | 90~95 | 90~95 | 90~95 |
