# Supplementary material for: Fishing capacity evaluation of fishing vessel based on cloud model
Source: Sci Rep. 2022 May 28;12:8976. doi: 10.1038/s41598-022-12852-8 (PMC9148315; doi:10.1038/s41598-022-12852-8)
Supplement: Supplementary file 3 — Supplementary Information 3. [file 41598_2022_12852_MOESM3_ESM.docx]

The following table is the evaluation of 32 experts on the importance of different indicators. The larger the value, the greater the importance.

|  | Specification | Fishing technology | Net | Resources and distribution of fishing objects |
| --- | --- | --- | --- | --- |
| Expert 1 | 0.875 | 0.625 | 0.125 | 0.375 |
| Expert 2 | 0.125 | 0.625 | 0.375 | 0.875 |
| Expert 3 | 0.625 | 0.375 | 0.875 | 0.125 |
| Expert 4 | 0.375 | 0.625 | 0.875 | 0.125 |
| Expert 5 | 0.875 | 0.625 | 0.375 | 0.125 |
| Expert 6 | 0.625 | 0.125 | 0.375 | 0.875 |
| Expert 7 | 0.375 | 0.875 | 0.625 | 0.125 |
| Expert 8 | 0.375 | 0.625 | 0.125 | 0.875 |
| Expert 9 | 0.875 | 0.125 | 0.625 | 0.375 |
| Expert 10 | 0.625 | 0.125 | 0.875 | 0.375 |
| Expert 11 | 0.375 | 0.625 | 0.125 | 0.875 |
| Expert 12 | 0.875 | 0.125 | 0.625 | 0.375 |
| Expert 13 | 0.625 | 0.125 | 0.375 | 0.875 |
| Expert 14 | 0.125 | 0.375 | 0.625 | 0.875 |
| Expert 15 | 0.875 | 0.625 | 0.375 | 0.125 |
| Expert 16 | 0.875 | 0.125 | 0.375 | 0.625 |
| Expert 17 | 0.875 | 0.375 | 0.625 | 0.125 |
| Expert 18 | 0.875 | 0.375 | 0.125 | 0.625 |
| Expert 19 | 0.875 | 0.375 | 0.625 | 0.125 |
| Expert 20 | 0.875 | 0.125 | 0.625 | 0.375 |
| Expert 21 | 0.125 | 0.375 | 0.625 | 0.875 |
| Expert 22 | 0.875 | 0.125 | 0.375 | 0.625 |
| Expert 23 | 0.625 | 0.375 | 0.125 | 0.875 |
| Expert 24 | 0.375 | 0.625 | 0.875 | 0.125 |
| Expert 25 | 0.375 | 0.875 | 0.625 | 0.125 |
| Expert 26 | 0.875 | 0.375 | 0.625 | 0.125 |
| Expert 27 | 0.875 | 0.375 | 0.625 | 0.125 |
| Expert 28 | 0.875 | 0.375 | 0.625 | 0.125 |
| Expert 29 | 0.875 | 0.375 | 0.625 | 0.125 |
| Expert 30 | 0.125 | 0.625 | 0.875 | 0.375 |
| Expert 31 | 0.625 | 0.875 | 0.375 | 0.125 |
| Expert 32 | 0.375 | 0.625 | 0.125 | 0.875 |

Table 1 Primary index

Table 2 Secondary index(Specification)

|  | Total power | Tonnage | Size | Age | Material |
| --- | --- | --- | --- | --- | --- |
| Expert 1 | 0.9 | 0.7 | 0.5 | 0.3 | 0.1 |
| Expert 2 | 0.7 | 0.9 | 0.5 | 0.3 | 0.1 |
| Expert 3 | 0.7 | 0.9 | 0.3 | 0.5 | 0.1 |
| Expert 4 | 0.9 | 0.7 | 0.5 | 0.3 | 0.1 |
| Expert 5 | 0.7 | 0.5 | 0.9 | 0.3 | 0.1 |
| Expert 6 | 0.5 | 0.3 | 0.9 | 0.7 | 0.1 |
| Expert 7 | 0.9 | 0.7 | 0.5 | 0.3 | 0.1 |
| Expert 8 | 0.5 | 0.9 | 0.7 | 0.3 | 0.1 |
| Expert 9 | 0.7 | 0.5 | 0.9 | 0.1 | 0.3 |
| Expert 10 | 0.7 | 0.9 | 0.5 | 0.1 | 0.3 |
| Expert 11 | 0.5 | 0.9 | 0.7 | 0.1 | 0.3 |
| Expert 12 | 0.7 | 0.9 | 0.5 | 0.1 | 0.3 |
| Expert 13 | 0.7 | 0.9 | 0.3 | 0.1 | 0.5 |
| Expert 14 | 0.9 | 0.7 | 0.3 | 0.1 | 0.5 |
| Expert 15 | 0.9 | 0.5 | 0.7 | 0.3 | 0.1 |
| Expert 16 | 0.9 | 0.1 | 0.7 | 0.5 | 0.3 |
| Expert 17 | 0.5 | 0.7 | 0.9 | 0.1 | 0.3 |
| Expert 18 | 0.5 | 0.9 | 0.7 | 0.1 | 0.3 |
| Expert 19 | 0.7 | 0.1 | 0.9 | 0.5 | 0.3 |
| Expert 20 | 0.5 | 0.3 | 0.7 | 0.1 | 0.9 |
| Expert 21 | 0.9 | 0.3 | 0.5 | 0.7 | 0.1 |
| Expert 22 | 0.9 | 0.5 | 0.7 | 0.1 | 0.3 |
| Expert 23 | 0.7 | 0.9 | 0.5 | 0.1 | 0.3 |
| Expert 24 | 0.9 | 0.5 | 0.3 | 0.7 | 0.1 |
| Expert 25 | 0.7 | 0.9 | 0.5 | 0.3 | 0.1 |
| Expert 26 | 0.7 | 0.3 | 0.5 | 0.1 | 0.9 |
| Expert 27 | 0.7 | 0.5 | 0.9 | 0.3 | 0.1 |
| Expert 28 | 0.3 | 0.5 | 0.1 | 0.7 | 0.9 |
| Expert 29 | 0.9 | 0.5 | 0.7 | 0.1 | 0.3 |
| Expert 30 | 0.3 | 0.9 | 0.7 | 0.5 | 0.1 |
| Expert 31 | 0.9 | 0.3 | 0.5 | 0.1 | 0.7 |
| Expert 32 | 0.5 | 0.3 | 0.7 | 0.1 | 0.9 |

Table 3 Secondary index(Net)

|  | Trawl | Purse net | Trap net | Gill net | Net size | Net main size | Number of nets | Net output | Cover net | Fishing tackle | Miscellaneous fishing gear |
| --- | --- | --- | --- | --- | --- | --- | --- | --- | --- | --- | --- |
| Expert 1 | 5.915 | 5.005 | 4.095 | 3.185 | 8.645 | 9.55 | 7.735 | 6.825 | 1.365 | 2.275 | 0.455 |
| Expert 2 | 7.735 | 6.825 | 5.915 | 5.005 | 8.645 | 0.455 | 1.365 | 9.55 | 4.095 | 3.185 | 2.275 |
| Expert 3 | 9.55 | 5.005 | 4.095 | 3.185 | 5.915 | 6.825 | 8.645 | 7.735 | 1.365 | 2.275 | 0.455 |
| Expert 4 | 7.735 | 6.825 | 5.915 | 5.005 | 9.55 | 8.645 | 2.275 | 1.365 | 4.095 | 0.455 | 3.185 |
| Expert 5 | 9.55 | 8.645 | 6.825 | 7.735 | 3.185 | 4.095 | 5.915 | 5.005 | 2.275 | 1.365 | 0.455 |
| Expert 6 | 3.185 | 5.915 | 2.275 | 4.095 | 8.645 | 7.735 | 6.825 | 9.55 | 5.005 | 1.365 | 0.455 |
| Expert 7 | 5.915 | 5.005 | 2.275 | 1.365 | 7.735 | 8.645 | 9.55 | 6.825 | 4.095 | 3.185 | 0.455 |
| Expert 8 | 8.645 | 9.55 | 7.735 | 6.825 | 0.455 | 1.365 | 3.185 | 2.275 | 5.915 | 5.005 | 4.095 |
| Expert 9 | 9.55 | 8.645 | 7.735 | 6.825 | 0.455 | 1.365 | 3.185 | 2.275 | 5.005 | 5.915 | 4.095 |
| Expert 10 | 9.55 | 7.735 | 5.915 | 8.645 | 3.185 | 0.455 | 2.275 | 1.365 | 5.005 | 6.825 | 4.095 |
| Expert 11 | 9.55 | 5.005 | 6.825 | 3.185 | 8.645 | 7.735 | 0.455 | 1.365 | 4.095 | 2.275 | 5.915 |
| Expert 12 | 5.005 | 3.185 | 4.095 | 5.915 | 7.735 | 8.645 | 6.825 | 9.55 | 1.365 | 0.455 | 2.275 |
| Expert 13 | 9.55 | 8.645 | 7.735 | 6.825 | 0.455 | 1.365 | 3.185 | 2.275 | 5.005 | 5.915 | 4.095 |
| Expert 14 | 4.095 | 7.735 | 5.915 | 8.645 | 0.455 | 1.365 | 3.185 | 2.275 | 6.825 | 9.55 | 5.005 |
| Expert 15 | 9.55 | 8.645 | 4.095 | 3.185 | 7.735 | 6.825 | 5.915 | 5.005 | 1.365 | 2.275 | 0.455 |
| Expert 16 | 5.915 | 5.005 | 4.095 | 3.185 | 7.735 | 9.55 | 8.645 | 6.825 | 2.275 | 0.455 | 1.365 |
| Expert 17 | 9.55 | 8.645 | 7.735 | 5.915 | 0.455 | 1.365 | 3.185 | 2.275 | 6.825 | 4.095 | 5.005 |
| Expert 18 | 8.645 | 7.735 | 6.825 | 5.915 | 0.455 | 1.365 | 3.185 | 2.275 | 5.005 | 4.095 | 9.55 |
| Expert 19 | 7.735 | 6.825 | 2.275 | 3.185 | 5.005 | 4.095 | 9.55 | 8.645 | 5.915 | 0.455 | 1.365 |
| Expert 20 | 9.55 | 8.645 | 7.735 | 5.005 | 2.275 | 3.185 | 0.455 | 1.365 | 6.825 | 4.095 | 5.915 |
| Expert 21 | 9.55 | 8.645 | 5.915 | 5.005 | 6.825 | 7.735 | 1.365 | 0.455 | 4.095 | 3.185 | 2.275 |
| Expert 22 | 9.55 | 8.645 | 7.735 | 6.825 | 0.455 | 1.365 | 3.185 | 2.275 | 5.915 | 5.005 | 4.095 |
| Expert 23 | 9.55 | 8.645 | 6.825 | 7.735 | 0.455 | 1.365 | 2.275 | 3.185 | 5.005 | 5.915 | 4.095 |
| Expert 24 | 5.915 | 5.005 | 4.095 | 7.735 | 0.455 | 1.365 | 3.185 | 2.275 | 8.645 | 9.55 | 6.825 |
| Expert 25 | 5.915 | 5.005 | 4.095 | 3.185 | 8.645 | 9.55 | 7.735 | 6.825 | 1.365 | 2.275 | 0.455 |
| Expert 26 | 7.735 | 5.005 | 5.915 | 6.825 | 9.55 | 8.645 | 1.365 | 0.455 | 4.095 | 2.275 | 3.185 |
| Expert 27 | 8.645 | 7.735 | 9.55 | 6.825 | 5.005 | 5.915 | 3.185 | 4.095 | 1.365 | 0.455 | 2.275 |
| Expert 28 | 5.915 | 5.005 | 3.185 | 4.095 | 9.55 | 6.825 | 7.735 | 8.645 | 1.365 | 2.275 | 0.455 |
| Expert 29 | 7.735 | 5.005 | 6.825 | 5.915 | 9.55 | 8.645 | 1.365 | 0.455 | 3.185 | 4.095 | 2.275 |
| Expert 30 | 9.55 | 5.005 | 2.275 | 8.645 | 6.825 | 5.915 | 0.455 | 1.365 | 7.735 | 4.095 | 3.185 |
| Expert 31 | 9.55 | 8.645 | 7.735 | 6.825 | 4.095 | 5.005 | 5.915 | 0.455 | 2.275 | 3.185 | 1.365 |
| Expert 32 | 6.825 | 5.915 | 5.005 | 4.095 | 8.645 | 9.55 | 1.365 | 7.735 | 3.185 | 0.455 | 2.275 |

Table 4 Secondary index(Fishing technology)

|  | Fish detection device | Ship-on machinery |
| --- | --- | --- |
| Expert 1 | 0.75 | 0.25 |
| Expert 2 | 0.25 | 0.75 |
| Expert 3 | 0.75 | 0.25 |
| Expert 4 | 0.75 | 0.25 |
| Expert 5 | 0.75 | 0.25 |
| Expert 6 | 0.75 | 0.25 |
| Expert 7 | 0.25 | 0.75 |
| Expert 8 | 0.25 | 0.75 |
| Expert 9 | 0.25 | 0.75 |
| Expert 10 | 0.25 | 0.75 |
| Expert 11 | 0.25 | 0.75 |
| Expert 12 | 0.25 | 0.75 |
| Expert 13 | 0.25 | 0.75 |
| Expert 14 | 0.25 | 0.75 |
| Expert 15 | 0.75 | 0.25 |
| Expert 16 | 0.25 | 0.75 |
| Expert 17 | 0.25 | 0.75 |
| Expert 18 | 0.75 | 0.25 |
| Expert 19 | 0.25 | 0.75 |
| Expert 20 | 0.75 | 0.25 |
| Expert 21 | 0.75 | 0.25 |
| Expert 22 | 0.25 | 0.75 |
| Expert 23 | 0.75 | 0.25 |
| Expert 24 | 0.25 | 0.75 |
| Expert 25 | 0.75 | 0.25 |
| Expert 26 | 0.25 | 0.75 |
| Expert 27 | 0.25 | 0.75 |
| Expert 28 | 0.75 | 0.25 |
| Expert 29 | 0.75 | 0.25 |
| Expert 30 | 0.75 | 0.25 |
| Expert 31 | 0.25 | 0.75 |
| Expert 32 | 0.75 | 0.25 |

Table 5 Secondary index(Resources and distribution of fishing objects)

|  | Fishery resources | Operating environment | Work time | Fishing period |
| --- | --- | --- | --- | --- |
| Expert 1 | 0.875 | 0.625 | 0.375 | 0.125 |
| Expert 2 | 0.875 | 0.125 | 0.375 | 0.625 |
| Expert 3 | 0.875 | 0.375 | 0.125 | 0.625 |
| Expert 4 | 0.875 | 0.375 | 0.625 | 0.125 |
| Expert 5 | 0.625 | 0.125 | 0.375 | 0.875 |
| Expert 6 | 0.875 | 0.375 | 0.125 | 0.625 |
| Expert 7 | 0.875 | 0.375 | 0.625 | 0.125 |
| Expert 8 | 0.875 | 0.125 | 0.375 | 0.625 |
| Expert 9 | 0.875 | 0.125 | 0.375 | 0.625 |
| Expert 10 | 0.875 | 0.125 | 0.625 | 0.375 |
| Expert 11 | 0.875 | 0.625 | 0.125 | 0.375 |
| Expert 12 | 0.875 | 0.375 | 0.125 | 0.625 |
| Expert 13 | 0.875 | 0.375 | 0.125 | 0.625 |
| Expert 14 | 0.875 | 0.125 | 0.375 | 0.625 |
| Expert 15 | 0.125 | 0.625 | 0.375 | 0.875 |
| Expert 16 | 0.875 | 0.375 | 0.125 | 0.625 |
| Expert 17 | 0.875 | 0.125 | 0.375 | 0.625 |
| Expert 18 | 0.625 | 0.375 | 0.125 | 0.875 |
| Expert 19 | 0.875 | 0.625 | 0.375 | 0.125 |
| Expert 20 | 0.125 | 0.625 | 0.875 | 0.375 |
| Expert 21 | 0.875 | 0.625 | 0.125 | 0.375 |
| Expert 22 | 0.625 | 0.375 | 0.125 | 0.875 |
| Expert 23 | 0.875 | 0.125 | 0.375 | 0.625 |
| Expert 24 | 0.875 | 0.375 | 0.125 | 0.625 |
| Expert 25 | 0.875 | 0.125 | 0.375 | 0.625 |
| Expert 26 | 0.375 | 0.125 | 0.875 | 0.625 |
| Expert 27 | 0.875 | 0.375 | 0.125 | 0.625 |
| Expert 28 | 0.875 | 0.375 | 0.125 | 0.625 |
| Expert 29 | 0.875 | 0.125 | 0.375 | 0.625 |
| Expert 30 | 0.875 | 0.125 | 0.375 | 0.625 |
| Expert 31 | 0.625 | 0.375 | 0.125 | 0.875 |
| Expert 32 | 0.875 | 0.375 | 0.125 | 0.625 |

The following table shows the evaluation matrix for each indicator, and the values are determined by the experts' scores for the importance of each indicator.

Table 6 Comparative criteria level matrix (Level 1 indicators)

| A_ij_ | B_1_ | B_2_ | B_3_ | B_4_ |
| --- | --- | --- | --- | --- |
| B_1_ | 1 | 3 | 2 | 3 |
| B_2_ | 1/3 | 1 | 1/2 | 1 |
| B_3_ | 1/2 | 2 | 1 | 2 |
| B_4_ | 1/3 | 1 | 1/2 | 1 |

Table 7 Comparative criteria level matrix (Level 2 indicators)

| A_ij_ | C_1_ | C_2_ | C_3_ | C_4_ | C_5_ |
| --- | --- | --- | --- | --- | --- |
| C_1_ | 1 | 2 | 2 | 4 | 3 |
| C_2_ | 1/2 | 1 | 2 | 3 | 2 |
| C_3_ | 1/2 | 1/2 | 1 | 4 | 4 |
| C_4_ | 1/4 | 1/3 | 1/4 | 1 | 1/3 |
| C_5_ | 1/3 | 1/2 | 1/4 | 3 | 1 |

Table 8 Comparative criteria level matrix (Level 2 indicators)

| A_ij_ | C_6_ | C_7_ |
| --- | --- | --- |
| C_6_ | 1 | 1/2 |
| C_7_ | 2 | 1 |

Table 9 Comparative criteria level matrix (Level 2 indicators)

| A_ij_ | C_8_ | C_9_ | C_10_ | C_11_ | C_12_ | C_13_ | C_14_ | C_15_ | C_16_ | C_17_ | C_18_ |
| --- | --- | --- | --- | --- | --- | --- | --- | --- | --- | --- | --- |
| C_8_ | 1 | 2 | 3 | 3 | 4 | 4 | 5 | 5 | 5 | 6 | 6 |
| C_9_ | 1/2 | 1 | 2 | 2 | 2 | 2 | 4 | 4 | 4 | 5 | 5 |
| C_10_ | 1/3 | 1/2 | 1 | 1 | 2 | 2 | 3 | 3 | 3 | 4 | 4 |
| C_11_ | 1/3 | 1/2 | 1 | 1 | 2 | 2 | 3 | 3 | 3 | 4 | 4 |
| C_12_ | 1/4 | 1/2 | 1/2 | 1/2 | 1 | 1 | 2 | 2 | 2 | 3 | 3 |
| C_13_ | 1/4 | 1/2 | 1/2 | 1/2 | 1 | 1 | 2 | 2 | 2 | 2 | 3 |
| C_14_ | 1/5 | 1/4 | 1/3 | 1/3 | 1/2 | 1/2 | 1 | 1 | 1 | 2 | 2 |
| C_15_ | 1/5 | 1/4 | 1/3 | 1/3 | 1/2 | 1/2 | 1 | 1 | 1 | 2 | 2 |
| C_16_ | 1/5 | 1/4 | 1/3 | 1/3 | 1/2 | 1/2 | 1 | 1 | 1 | 2 | 2 |
| C_17_ | 1/6 | 1/5 | 1/4 | 1/4 | 1/3 | 1/2 | 1/2 | 1/2 | 1/2 | 1 | 2 |
| C_18_ | 1/6 | 1/5 | 1/4 | 1/4 | 1/3 | 1/3 | 1/2 | 1/2 | 1/2 | 1/2 | 1 |

Table 10 Comparative criteria level matrix (Level 2 indicators)

| A_ij_ | C_19_ | C_20_ | C_21_ | C_22_ |
| --- | --- | --- | --- | --- |
| C_19_ | 1 | 3 | 3 | 2 |
| C_20_ | 1/3 | 1 | 2 | 1/2 |
| C_21_ | 1/3 | 1/2 | 1 | 1/3 |
| C_22_ | 1/2 | 2 | 3 | 1 |
